# Obstetrical Constraints and the Origin of Extended Postnatal Brain Maturation in Hominin Evolution

**DOI:** 10.3390/biology13060398

**Published:** 2024-05-31

**Authors:** Pierre Frémondière, Martin Haeusler, Lionel Thollon, Nicole M. Webb, François Marchal

**Affiliations:** 1 Faculty of Medical and Paramedical Sciences, School of Midwifery, Aix Marseille University, 51 Boulevard Pierre Dramard, 13344 Marseille CEDEX 15, France; 2 UMR 7268 ADES, Aix Marseille University, EFS, CNRS, 51 Boulevard Pierre Dramard, 13344 Marseille CEDEX 15, France; francois.marchal@univ-amu.fr; 3 Institute of Evolutionary Medicine, University of Zürich, Winterthurerstrasse 190, 8057 Zürich, Switzerland; martin.haeusler@iem.uzh.ch; 4 LBA, Aix Marseille University, Gustave Eiffel University, 51 Boulevard Pierre Dramard, 13344 Marseille CEDEX 15, France; lionel.thollon@univ-eiffel.fr; 5 Department of Palaeoanthropology, Senckenberg Gesellschaft für Naturforschung, Senckenberganlage 25, 60325 Frankfurt am Main, Germany

**Keywords:** secondary altriciality, dystocia, australopithecines, cephalo-pelvic disproportion, human evolution

## Abstract

**Simple Summary:**

The unique complexity of human childbirth is traditionally attributed to the opposing selection pressures of bipedal locomotion and large brains. Here, we explore this trade-off in *Australopithecus* with canonical discriminant analyses using different fetal head sizes. We reveal that the shape of the pelvis in *Australopithecus* led to a tight fit between the mother’s pelvis and the newborn head despite their relatively small brain sizes. To alleviate this obstetrical dilemma, australopithecines must have already given birth to secondarily altricial infants that were helpless at birth like those of extant humans. Cognitive development and some aspects of the modern life history pattern therefore likely originated prior to the appearance of the genus *Homo*.

**Abstract:**

The origin of difficult birth is still a matter of debate in obstetrics. Recent studies hypothesized that early hominins already experienced obstructed labor even with reduced neonatal head sizes. The aim of this work is to test this hypothesis using an extant obstetrical sample with known delivery outcomes. Three delivery outcomes (i.e., instrument-assisted, Caesarean section, and vaginal birth) were evaluated using a discriminant analysis based on 131 mother–baby dyads and 36 feto-pelvic variables. This obstetrical sample was compared with 20 australopithecine “dyads” generated from the combination of six pelvic reconstructions (three for *Australopithecus afarensis*, two for *A. africanus,* and one for *A. sediba*) and three fetal head size estimations. The obstetrical analysis revealed that dystocic births can be predicted by pelvic features such as an anteroposteriorly flattened pelvic inlet. Australopithecines shared these pelvic morphologies with humans and had eutocic birth only for infants of 110 g brain size or smaller, equaling a human-like neonatal/adult brain size ratio of 25–28%. Although birth mechanism cannot be deduced, the newborn/adult brain size ratio was likely more human-like than previously thought, suggesting that australopithecines were secondarily altricial to circumvent instances of obstructed labor and subsequently require a prolonged postnatal brain growth period, implying some aspects of life history pattern similar to modern humans.

## 1. Introduction

The diversity of life history traits among mammals reflects unique strategies for ensuring optimal growth, efficient reproduction, raising offspring to independence, and increasing maximal life span within certain ecological constraints [1]. Among the different traits attributed to the overall life history of the species, several perinatal features, e.g., gestation length, postnatal ontogeny, and duration of breastfeeding, are shaped by natural selection to optimize the survival of the mother and infant, thereby advancing reproductive success [2,3].

In mammals, these perinatal patterns are typically thought to fall into the following two distinct strategies: altriciality and precociality [4]. Altricial species usually give birth after a short gestation to neonates without hair, with closed eyes and not fully developed ears, and without locomotor abilities. This represents the primitive condition of mammals and makes the newborn very dependent on the mother [5]. On the contrary, in precocial species, neonates need less life-sustaining care from parents and have well-developed sensory organs [6]. Precociality has been developed independently in different mammal lineages, and these species generally have longer gestation and small litters. Humans are generally precocial, sharing with other primates an extended gestational period and a large adult brain size, and they usually give birth to a single offspring; however, they have a small neonate/adult brain size ratio and a helpless newborn typical of altricial species [7]. This secondary, partial reversal to the primitive life history pattern has been referred to as “secondary altriciality” [4,7]. For parents, this requires a higher investment, which has the following cultural and biological implications: the task of childcare requires the support of other members of the group [8], and the mother’s milk is adapted for sustaining rapid brain growth during early infancy [3].

When compared with that of other hominoids, birth in extant humans is markedly distinct [9,10] and characterized by a complex birth pattern: the fetus has to flex and rotate its head to ensure the descent through the convoluted birth canal [9]. This complex birth mechanism is due to the contorted shape of the maternal pelvis. Since the upper part (i.e., inlet level) of the birth canal is shortened in the anteroposterior dimension, the fetus has to enter it in a transverse or oblique head orientation. However, at the middle and lower part of the birth canal, the *levator hiatus* is sagittally elongated and the midplane and outlet are anteroposteriorly expanded, so the fetus has to rotate in a sagittal head position [11]. Besides the complex birth process, humans tend to have longer births and are at a higher risk of labor difficulties (i.e., dystocic labor, from Greek *dys*: difficult and *tokos*: birth; contra eutocic labor: easy birth) [12]. The global rate of obstructed labor is estimated to be 3–6% [13], but the etiopathology of obstructed labor remains poorly understood. 

In clinical practice, the female pelvis has been investigated in order to predict dystocic labor [14,15,16,17]. Indeed, the prediction of dystocic birth would help to avoid unnecessarily prolonged labor and potentially eliminate difficulty during delivery for both the mother and the fetus by proposing a Caesarean section before the onset of labor [15]. Typically, six diameters of the pelvis [14,15] and several fetal variables [16] are used for this type of prediction. However, when used alone, the pelvic dimensions or the estimated neonatal weight are usually not sufficient to accurately predict delivery outcomes [16]. As such, some clinical studies also consider the head circumference, the biparietal diameter, the abdominal circumference, and the abdominal transverse diameter in addition to pelvic dimensions [16]. Even if the clinical tools (i.e., scoring systems based on pelvic and fetal measurements) help in predicting dystocic labor, they have proven to be inadequate in a low-risk population [17], suggesting that the feto-pelvic constraint has to be investigated more rigorously in order to understand and improve delivery outcomes via pelvimetry. 

Several hypotheses assume that human pelvic shape is also influenced by evolutionary forces driven by the thermal environment [18], genetic drift [19], and ecological stress [20] and that these factors could aid in elucidating the source of the marked pelvic sexual dimorphism in the human pelvis [21,22]. When and how these pressures shaped extant human birth remains an open and debated question amongst evolutionary biologists and clinicians [10,20,23,24]. Bipedalism is believed to be among the most influential factors as hominins have undergone a drastic restructuring of pelvic morphology to facilitate optimal efficiency during our unique upright locomotion [25,26]. Further, the reduction in critical diameters such as the distance between the sacroiliac joint and the hip joint, as an adaptation to bipedal locomotion, may have influenced birth by altering obstetric measures within the pelvis [27]. Encephalization may have contributed to this by further reducing the space allocated to the bony birth canal [28], especially since brain size increases substantially over the course of human evolution [29]. This conflict between pelvic adaptations to accommodate a large fetal brain size and selection pressures to reduce the anteroposterior diameter of the pelvis because of bipedal adaptation has been coined the “obstetrical dilemma” [30]. One solution to this conflict is to give birth at an earlier stage of development, when the large head of the human neonate can still pass through the birth canal [31], and human infants are therefore secondarily altricial. This results in a low ratio of neonatal/adult brain size in humans. Modern humans have a neonatal brain size that is 28% of the adult brain size, while this ratio is 43% in non-human primates [5]. To investigate this dilemma among early hominins, the australopithecines are interesting because they have a reduced anteroposterior diameter of the pelvis as modern humans combined with a small adult brain size, similar to great apes [32], and a large range of pelvic reconstructions is available.

In order to test the hypothesis that early hominins already experienced obstructed labor even with reduced neonatal head sizes [11], in this study, we aim to investigate the birth process in different australopithecine “dyads” and explore their delivery outcomes. A dyad is a combination of a set of neonatal dimensions with a set of pelvic measurements. The australopithecine ”dyads” were constructed by combing the published pelvic reconstructions with three different neonatal head sizes based on a 180 g brain mass, as predicted from a non-human primate model, a 110 g brain mass, as predicted from a modern human model, and an intermediate brain mass of 145 g [11]. The same set of neonatal dimensions and pelvic measurements is recorded for extant human dyads with known delivery outcomes. This approach offers insights into how these measures of the fetus and maternal pelvis interact and ultimately affect birth outcomes. These delivery outcomes are evaluated using a discriminant analysis based on the modern obstetrical sample. Then, the australopithecine “dyads” are included subsequently in the analyses to determine their likely delivery outcomes.

## 2. Materials and Methods

### 2.1. Clinical Data

One hundred and thirty-one women at Saint Joseph Hospital, Marseille, France, were recruited from 29 March 2011 to 10 December 2013 for this single-center study. The inclusion criteria were birth at term with the fetus in a cephalic presentation. Exclusion criteria were Caesarean deliveries performed in cases of abnormal fetal heart rate or before 2 h of the arrest of labor, abnormal uterine contraction, twin pregnancies, and iterative Caesarean sections. The 131 deliveries included in this study were spontaneous vaginal delivery in 51 cases, instrument-assisted delivery in 56, and Caesarean section for the arrest of labor in 24 cases. All 131 women had both epidural anesthesia and a pelvic CT scan, i.e., radiological measurement of the parameters of the pelvis, before delivery. Indications for a pelvic CT were a scarred uterus, a breech presentation (but cephalic presentation at the beginning of labor), and suspicion or a history of feto-pelvic disproportion. All patients enrolled in this study had the benefits/risks and long-term risks of CT scanning explained and all gave their consent for the scanning. This study was approved by the South Mediterranean II Ethical Committee for the Protection of Persons (local ethics committee number: 1d-RCB 2011-A00072-39), and written informed consent was obtained from all the patients.

**Figure 1:**
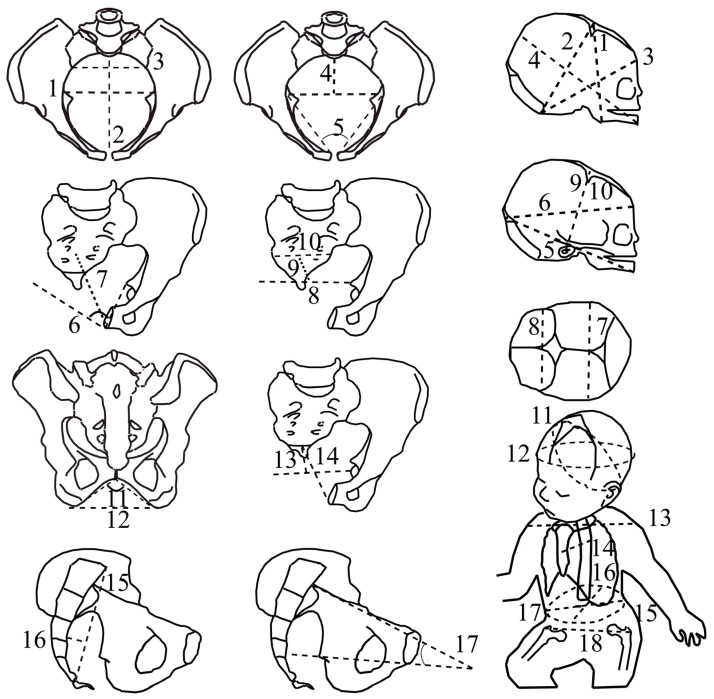
The feto-pelvic variables of the extant obstetrical and fossil samples. Pelvic variables: (1) maximal transverse diameter of the inlet; (2) inlet antero-posterior diameter, (3) inlet sacral breadth, (4) posterior inlet diameter, (5) pectineal angle (6) midplane angle, (7) midplane antero-posterior diameter, (8) interspinous diameter, (9) posterior midplane diameter, (10) midplane sacral breadth, (11) subpubic angle, (12) transverse outlet diameter, (13) posterior outlet diameter, (14) outlet antero-posterior diameter, (15) sacral chord length, (16) sacral chord subtense, (17) inlet–midplane angle (angle formed by the inlet plane and the midplane, measured in a strict sagittal view). Fetal variables: (1) submentobregmatic diameter, (2) suboccipitobregmatic diameter, (3) suboccipitofrontal diameter, (4) mentovertical diameter, (5) mento-occipital diameter, (6) occipitofrontal diameter, (7) biparietal diameter, (8) bitemporal diameter (9) right tragion–bregma diameter, (10) left tragion–bregma diameter, (11) suboccipitobregmatic circumference, (12) head circumference, (13) biacromial diameter, (14) sternum–thoracic vertebral diameter, (15) abdominal circumference, (16) abdominal sagittal diameter, (17) transverse abdominal diameter, (18) bitrochanterian diameter.

CT scans were performed with a 16-slice Siemens SOMATOM Definition Flash strip scanner located in the Medical Imaging Department of our hospital. The intersection gap was 0.6–1 mm. All pelvic diameters were measured with Amira software 5.0.0 (Thermo-Fisher Scientific, Merignac, France) by the same operator. A total of 17 pelvic variables were considered (Figure 1, Table 1). The newborn measurements were performed by the same operator during the postpartum period (on the first day of life, using anthropometric tools including a cephalometric compass, a tape measure, and a newborn scale). Nineteen fetal variables were measured (Figure 1, Table 1).

### 2.2. Statistical Analyses

Canonical discriminant analyses were used to identify relevant variables in the prediction of feto-pelvic disproportion. This is commonly performed to statistically separate more than 2 groups, here, spontaneous vaginal birth, Caesarean delivery, and instrument-assisted delivery, by simultaneously using a large number of predictors common to these groups, here, the feto-pelvic variables. The importance of each feto-pelvic variable was assessed by the standardized coefficient of the discriminant function (SCC). The significance of the difference between the centroid of each group was tested using the lambda of Wilks (λ Wilks). A *p*-value of <0.05 was considered statistically significant. Statistical tests were performed using SPSS Statistics (IBM).

### 2.3. Pelvic Meshes

**Figure 2:**
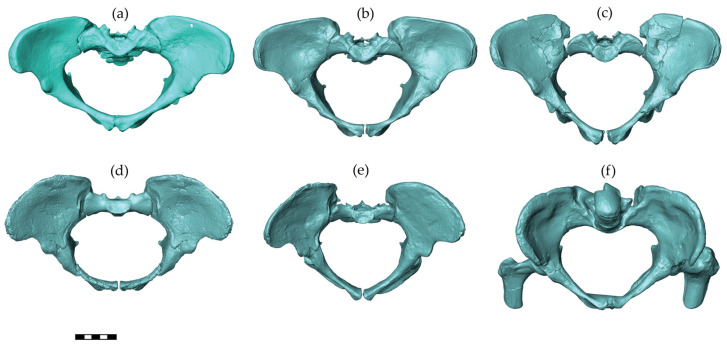
Australopithecine pelvic reconstructions used in this study. From the left to right and top to bottom, these reconstructions are A.L. 288-1 (*Australopithecus afarensis*): by (**a**) Tague and Lovejoy [10], (**b**) Haeusler and Schmid [46], and (**c**) Brassey et al. [47]; Sts 14 (*A*. *africanus*): (**d**) Berge and Goularas [9] and (**e**) Haeusler and Schmid [46]; and MH2 (*A*. *sediba*): (**f**) Kibii et al. [43]. All pelves are shown in a view perpendicular to the pelvic inlet. The scale bar is 5 cm.

Australopithecines are early hominins that lived in South and East Africa between 4 and 2 million years ago [40]. The pelvic reconstructions of three australopithecine species were considered in this study, including *Australopithecus afarensis*, *A. africanus*, and *A. sediba* (Figure 2). Assuming the same pelvic sexual dimorphism in australopithecines and modern humans and based on associated dental morphology of these early hominins, reliable attribution of the sex is possible among these pelvises [41,42,43]. The A.L. 288-1 (Hadar, Ethiopia, 1974) pelvis belongs to a female *A. afarensis* dated to around 3.2 million years [44] with an individual age at death of close to the end of the third decade of modern humans [45]. For this pelvis, we included three different reconstructions in the analyses. The manual reconstruction of Haeusler and Schmid [46] was scanned with a high-resolution surface scanner (PT-M4c, Polymetric GmbH, Darmstadt, Germany). Lovejoy’s manual reconstruction [42] was generously provided by the author as a 3D surface scanner-generated model based on a cast. We scaled the 3D model of Lovejoy’s reconstruction by a factor of 1.046 sagittally and 1.033 mediolaterally as well as superoinferiorly to obtain the dimensions published by Tague and Lovejoy [10]. The virtual reconstruction of the A.L. 288-1 pelvis by Brassey et al. [47] was available as a 3D model from Figshare (https://doi.org/10.6084/m9.figshare.c.3462618 accessed on 12 February 2018). The Sts 14 pelvis (Sterkfontein, South Africa) is a presumed female *A. africanus* [48], with an age at death around 16 years compared with modern human standards [49], and it is dated to around 2.1–2.6 million years [50]. Two different reconstructions of Sts 14 were included as follows: the manual reconstruction by Haeusler and Schmid [46] was scanned with a PT-M4c, Polymetric GmbH, Darmstadt, Germany, whereas the virtual reconstruction by Berge and Goularas [9] was generously provided by one of the authors. The MH2 (Malapa, South Africa) pelvis is attributed to *A. sediba* [43] and dated to 2–1.8 million years [51]. The pelvis belongs to a female of advanced age (based on the heavily worn molars) [51]. For this pelvis, a cast of the reconstruction performed and provided by Schmid [43] was scanned with a PT-M4c high-resolution surface scanner.

### 2.4. Fetal Model

The fetal model used for the australopithecine “dyads” was based on a medical CT scan of a human fetus at 35 weeks of gestation. The CT images were segmented in Mimics 12.3 (https://www.materialise.com, accessed on 5 April 2013). Using the scaling relationship of neonatal-to-adult brain size based on 27 primate species, the mean neonatal brain mass for *A. afarensis*, *A. africanus*, and *A. sediba* is estimated to a range of 166–184 g [11]. In contrast, using the ratio typical of modern humans, a mean neonatal brain size of between 111 and 121 g is predicted for *Australopithecus*. Therefore, the fetal head model was scaled to conform to the brain masses of 110 g and 180 g as well as an intermediate brain mass of 145 g, using fetal neurocranial proportions (proportions of the cranial length and breadth) of chimpanzees and humans. This yielded fetal heads with biparietal diameters of 75 mm, 70 mm, and 64 mm, respectively, and occipito-frontal diameters of 87 mm, 81 mm, and 75 mm, respectively. The abdomen, thorax, shoulder, and hips of the fetus were scaled following the same process (i.e., scaled to 86%, 81%, and 75%), and the somatic fetal variables were measured with Amira 5.0.0 (Thermo-Fisher Scientific). Birthweight was estimated using the most relevant equation based on our dataset of fetal variables. The eighteen fetal variables, from the submentobregmatic to the bitrochanteric diameter (Figure 1), were considered the predictors, and birthweight was the dependent predictive variable. We used the coefficient of determination (r^2^) to determine the best predictive equation. The most appropriate fetal variable was the bitrochanteric diameter according to the following equation: birthweight (in gram) = 55.4 × bi-trochanteric diameter (in mm)—1548 (r^2^ = 0.868, SE = 328). This gives birthweights of 1900 g, 1700 g, and 1450 g associated with a brain size of 180 g, 145 g, and 110 g, respectively.

### 2.5. Inclusion of Fossil Dyads in the Canonical Discriminant Analyses

The same feto-pelvic variables collected for extant human dyads were measured in the australopithecine “dyads”. Then, the australopithecine “dyads” were added to the canonical discriminant analyses as supplementary individuals. The predicted group membership of the australopithecine “dyads” (i.e., spontaneous vaginal, instrument-assisted delivery, Caesarean section) was then calculated. In the reconstruction of Sts 14 of Berge and Goularas [9], the caudal sacral vertebrae were not restored. Therefore, a supplementary canonical discriminant analysis was performed with the removal of the following variables, which cannot be measured on the reconstruction: midplane antero-posterior diameter, midplane sacral breadth, posterior midplane diameter, posterior outlet diameter, and antero-posterior outlet diameter. The sacral chord length and sacral chord subtense, as defined by Schaal et al. [33], were also excluded from this supplementary canonical discriminant analysis.

## 3. Results

**Figure 3:**
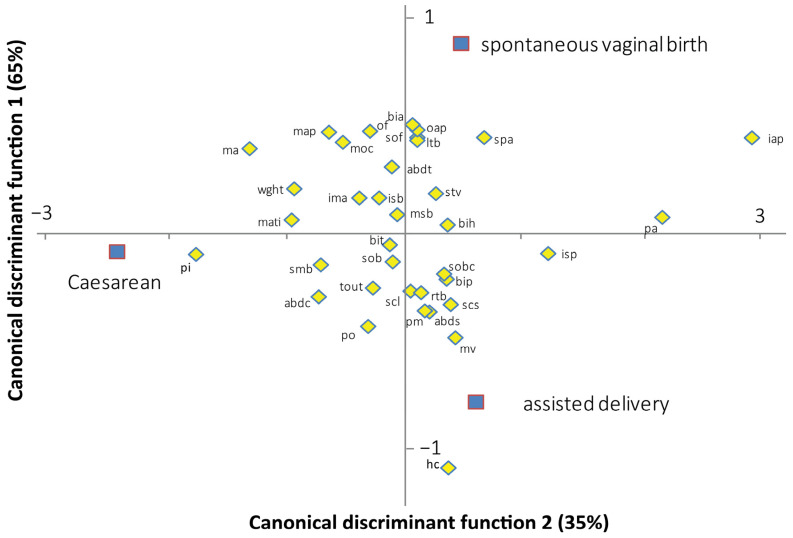
Standardized canonical coefficients of the fetal-pelvic variables between the two canonical discriminant functions, based on modern human data. mati: maximal transverse diameter of the inlet; pi: posterior inlet diameter; pa: pectineal angle; isb: inlet sacral breadth; iap: inlet antero-posterior diameter; map: midplane antero-posterior diameter; isp: interspinous diameter; msb: midplane sacral breadth; pm: posterior midplane diameter; ma: midplane angle; tout: transverse outlet diameter; spa: subpubic angle; po: posterior outlet diameter; oap: outlet antero-posterior diameter; ima: inlet–midplane angle; scl: sacral chord length; scs: sacral chord subtense; smb: submentobregmatic diameter; sob: suboccipitobregmatic diameter; sof: suboccipitofrontal diameter; mv: mentovertical diameter; moc: mento-occipital diameter; of: occipitofrontal diameter; bip: biparietal diameter; bit: bitemporal diameter; rtb: right tragion–bregma diameter; ltb: left tragion–bregma diameter; sobc: suboccipitobregmatic circumference; bia: biacromial diameter; stv: sternum–thoracic vertebral diameter; abdc: abdominal circumference; abds: abdominal sagittal diameter; abdt: transverse abdominal diameter; bih: bitrochanterian diameter; wght: birthweight.

The canonical discriminant analysis provides an assessment of the most important discriminant variables. Figure 3 shows the contribution of the feto-pelvic variables to the discrimination of the three delivery outcomes in the modern human dyads. Axis 1 discriminates the Caesarean sections from the spontaneous vaginal births and instrument-assisted deliveries. Axis 2 discriminates the spontaneous vaginal births from the instrument-assisted deliveries. Axes 1 and 2 explain 65% and 35% of the total variation, respectively. Of the 17 pelvic variables, the inlet antero-posterior diameter represents the most important variable for discriminating Caesarean sections from spontaneous vaginal and instrument-assisted deliveries (standardized coefficient of the first function, SCC1 = 2.406). Of the five variables of the pelvic inlet, three were oriented toward the Caesarean section group including the maximum transverse inlet diameter (SCC1 = −0.781), the posterior inlet diameter (SCC1 = −1.443), and the inlet sacral breadth (SCC1 = −0.175). Of the 19 fetal variables, the head circumference represents the most important variable for discriminating the spontaneous vaginal from instrument-assisted delivery (standardized coefficient of the second function, SCC2 = −1.024). Birthweight was oriented toward the Caesarean group (SCC2 = −0.763). The convoluted shape of the birth canal moderately contributed to the discrimination of the three delivery outcomes (inlet–midplane angle: SCC1 = −0.250; SCC2 = 0.140, sacral chord subtense: SCC1 = 0.320; SCC2 = −0.307).

Table 2 presents the classification error of the canonical discriminant analysis. Of the 24 women with Caesarean delivery, 19 (79.2%) were well-predicted. Of the remaining 107 women (i.e., without Caesarean delivery), 76 (71.0%) were well-predicted. There was significant discrimination between Caesarean delivery and the other delivery outcomes (λ Wilks = 0.345; *p* < 0.001). Of the 51 women with spontaneous vaginal birth, 32 (62.7%) were well-predicted, and of the 56 women with instrument-assisted delivery, 44 (78.6%) were well-predicted. The discrimination between vaginal birth and instrument-assisted delivery was not significant (λ Wilks = 0.659; *p* = 0.124).

**Figure 4:**
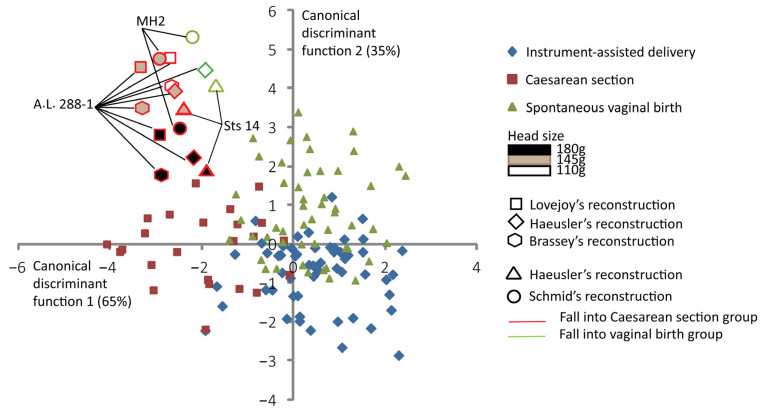
Canonical scores of the two canonical discriminant functions (CDFs) of instrument-assisted delivery, Caesarean section, and spontaneous vaginal birth, with australopithecine “dyads” as supplementary individuals. All the 131 extant human mother–baby dyads are represented with a color code corresponding to their obstetrical outcomes as follows: blue for instrument-assisted deliveries, red for Caesarean sections, and green for spontaneous vaginal births. Australopithecine “dyads” that are predicted to fall into the vaginal birth group are outlined in green. Those predicted to fall into the Caesarean section group are outlined in red.

Figure 4 shows the discrimination among the three delivery outcomes and the predictive classification of the australopithecine “dyads”. According to this predictive classification, the A.L. 288-1 pelvic reconstructions of Lovejoy [10] and Brassey et al. [47] are predicted to fall into the Caesarean section outcome for all brain sizes considered (Table 3). The A.L. 288-1 and the Sts 14 pelvis reconstructions of Haeusler and Schmid [46] are predicted to fall into the “vaginal birth” outcome with a brain size of 110 g. The MH2 reconstruction [43] is predicted to fall into the “vaginal birth” outcome with a brain size of 110 g (Table 3).

**Figure 5:**
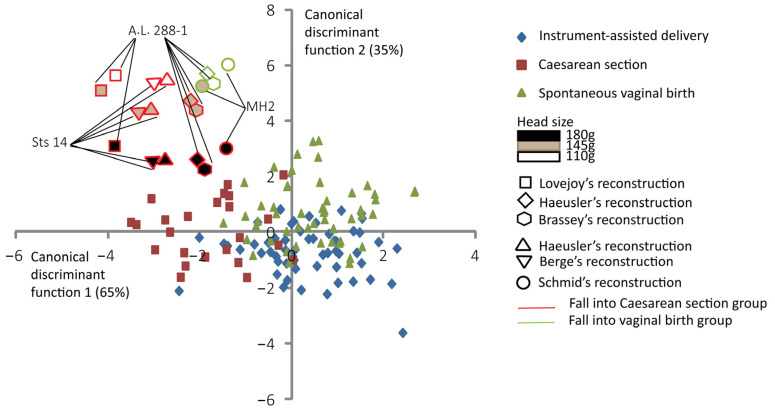
Canonical scores of the two canonical discriminant functions (CDFs) of instrument-assisted delivery, Caesarean section, and spontaneous vaginal birth, with the reconstruction of Berge and Goularas [9] and other australopithecine “dyads” as supplementary individuals. All the 131 extant human mother–baby dyads are represented with a color code corresponding to their obstetrical outcomes as follows: blue for instrument-assisted deliveries, red for Caesarean sections, and green for spontaneous vaginal births. Australopithecine “dyads” that are predicted to fall into the vaginal birth group are outlined in green. Those predicted to fall into the Caesarean section group are outlined in red.

Figure 5 shows the supplementary canonical discriminant analysis with the reconstruction of Sts 14 of Berge and Goularas [9], which is predicted to fall into the “Caesarean section” group with all brain sizes considered. In this analysis, the A.L. 288-1 reconstruction of Lovejoy [10] and the Sts 14 reconstruction of Haeusler and Schmid [46] are all predicted to fall in the “Caesarean delivery” group. The MH2 reconstruction of Schmid [43] is predicted to fall into the “Caesarean delivery” group for a brain size of 180 g. For this supplementary analysis, the overall prediction error is 68.7%.

## 4. Discussion

### 4.1. Discriminating Delivery Outcomes with Pelvic and Fetal Variables

Previous works in modern humans have attempted to predict delivery outcomes accurately based on feto-pelvic variables. Among these studies, Morgan and Thurnau [16] found the most reliable method (i.e., the fetal pelvic index) in determining the presence or absence of feto-pelvic disproportion. They found that 53 of 73 patients who required operative intervention were well-predicted (72.6%) and 62 identifications of 64 patients with spontaneous vaginal delivery were correctly identified (96.8%) [16]. However, Korhonen et al. [17] used the fetal pelvic index in a larger cohort (n = 966) and raised many doubts about the usefulness of this index. They found that 574 of 700 women with spontaneous vaginal (82%) and 170 of 233 women with Caesarean delivery (63.9%) were accurately identified. The sensitivity and specificity were low even when different cut-off values of the fetal pelvic index were considered (sensibility: 0.19–0.63; specificity: 0.66–0.97). In our study, the canonical discriminant functions provide comparable results to previous studies [16,17]. The canonical discriminant analyses significantly discriminate Caesarean section vs. non-Caesarean section groups (79.2% and 71.0% for women with and without Caesarean delivery), which is because Caesarean delivery resulted from inlet arrest in the case of feto-pelvic obstruction [52]. The inlet level is a virtually undeformable bony ring and is therefore particularly prone to feto-pelvic incongruence [36]. Accordingly, this dystocia is well-predicted with the bony pelvic variables. The midplane level is composed of soft tissues and is the location of the pelvic floor. These muscle and soft tissue components increase resistance against fetal descent [53] but are not considered in models reduced to only bony variables. This would explain the lack of clear discrimination between spontaneous vaginal and instrument-assisted birth and the important overlap between these two groups in this study. It is therefore difficult to identify a risk of mid-arrest for the australopithecine “dyads”. More studies are required, specifically involving pelvic floor modelization of australopithecines, to identify the potential for midplane obstruction in early hominins.

### 4.2. Specific Pelvic Pattern Related to Inlet Arrest

Among the women included in this study, those having a small antero-posterior inlet and a large maximum transverse inlet are more at risk of Caesarean delivery. This combination of pelvic features is associated with a flattened pelvis, which is the source of dystocic labor [54]. However, the convoluted shape of the birth canal, i.e., a high inlet–midplane angle, sacral chord length, and subtense, do not explain Caesarean deliveries. The human-pronounced curvature of the birth canal may be associated with the obstetric mechanism [9] rather than dystocic labor. In our results, australopithecine “dyads” are on the left side of axis 1, which is that of Caesarean sections (see Figure 4 and Figure 5). At the same time, they are shifted toward vaginal deliveries, following axis 2 in the positive direction. This shifting in the australopithecine “dyads” on axis 2 could be explained by the association with each pelvic reconstruction of identically reduced fetal dimensions for each dyad. These pelvic reconstructions share morphologic traits with modern humans, such as the sacrum and the pubic symphysis at the same level [10], the protrusion of the ischial spines [55], the large subpubic angle as well as an intermediate form between the non-human primate straight pathway, and the curved birth trajectory of modern humans [9]. In comparison to the human and non-human primate pelvis, australopithecines also exhibited some peculiar pelvic features such as the transversally oval pelvic inlet [9,10]. For example, for A.L. 288-1, the inlet index (i.e., obstetric conjugate / transverse inlet diameter × 100) is outside the range of variation in modern human females (57.6% in A.L. 288-1 vs. 77.6% in modern humans) [10]. This peculiar shape of the pelvic inlet could explain the important obstetric constraint observed in this study for australopithecine “dyads”.

### 4.3. Implication for Life History in Early Hominins

Independent of the reconstruction considered, we found that most australopithecine “dyads” are eutocic for a fetal brain size of 110 g. Dyads with a 180 g brain size are systematically dystocic. This would suggest that the pelvic reconstruction considered has a minimal impact on obstetrical deduction. These results are consistent with a previous study based on finite element and in silico simulation [11] in which it was suggested that only a 110 g fetal head size successfully passed through the bony pelvic of australopithecines. With this very different and completely independent method, based on a clinical rather than biomechanical approach, we found a similar estimation of the brain size at birth for australopithecines. Previous attempts to elucidate hominin birth mostly relied on single pelvic reconstructions to generate inferences about the obstetrics of australopithecines, such as *Australopithecus afarensis* [56], *A. africanus* [9,56], and *A. sediba* [57]. In fact, only three female individuals had a sufficiently complete set of coxal and sacral bones to propose a reconstruction of the pelvis including Sts 14, A.L. 288-1, and MH2 (Sts 65 has previously also been used for obstetrical analyses [58], but most likely belongs to a male individual [48] and at best allows the reconstruction of the inlet, although it does not have a sacrum). The aim of this work is to consider all the available pelvic reconstructions for *Australopithecus*. Although these three individuals might not represent the entire variability encompassed within this genus, the constitution of several dyads allows us to explore a range of likely possibilities, thus offering a robust approach despite the aforementioned limitations. Moreover, pelvic shape, obstetric capacity, and inferred neonatal head size are remarkably uniform in these three female australopithecines that belong to three different species spanning about 1.3 million years, suggesting that our results can probably be generalized for the genus *Australopithecus.* This method provides a better alternative than confining the paleo-obstetrical interpretations to a single individual, which would suffer from bias introduced by the reconstructions and the disparate variation noted across this genus. Therefore, *Australopithecus* probably gave birth to neonates with brain sizes of ca. 110 g, smaller than 145 g, 155 g (the brain size of a chimpanzee at birth), and 180 g. When adult brain sizes are considered for australopithecines, between 396 g and 432 g [11], the ratio of neonatal/adult brain size is between 25% and 28% for a brain size at birth of 110 g. In comparison, great apes have a brain size at birth that is 40–43% of adult brain size, while this ratio is 28% in modern humans [5,59]. This small brain size at birth should have been associated, like in modern humans, with a prolonged period of intensive brain growth and important neurological development, yet involving a considerable parental investment. This period after birth would have provided the basis for the cognitive development of the infant. The significant prolonged postnatal brain growth can only be sustained by the enrichment of lipids in the mother’s milk, which may represent a metabolic cost for the mother [3]. The “helpless”, secondarily altricial state at the birth of human newborns requires support provided by the mother, the parents, and finally, the whole human group [8]. Although it requires a substantial investment, this support implies important cognitive abilities from the mother and other caregivers to identify the precise needs of the infant [6]. Similar to other cooperative breeders, human parents share the costs associated with carrying infants [8]. Given the estimated brain size at birth in australopithecines, newborns were probably “helpless”, implying the presence of a cooperative breeding system, while a prolonged period and/or a fast brain growth rate were likely already present in these early hominins. This is consistent with previous studies suggesting an ape-like brain organization and protracted brain growth in *A. afarensis* [32]. This study focuses on the risk of obstructed labor, and it is not possible to make inferences about birth mechanism (i.e., rotational birth). Indeed, these mechanisms probably involve the pelvic floor muscles [11], and their configuration is not investigated in the present study. Nevertheless, further studies involving pelvic floor modelization of australopithecines are required to determine whether birth was rotational in these early hominins.

## 5. Conclusions

Our analyses revealed that australopithecines probably gave birth to infants with an estimated neonatal brain size of 110 g. This small brain size at birth decreases the ratio of neonatal/adult brain size. This would have been associated with significant postnatal neurological growth involving a significant degree of parental investment. Larger brain size at birth would have been a risk in childbirth, probably leading to increased instances of inlet arrest. Among the morphological features of the australopithecine pelvis, the flattened shape of the birth canal could explain the higher risk of obstruction for head sizes comparable to those predicted for non-human primates. This relatively small brain has implications for the handling of children in the early stages of life: the consequently prolonged brain growth places a considerable burden on the parents, especially the mother, whose metabolic costs would have been reallocated during breastfeeding. To alleviate this burden, a cooperative breeding strategy could have evolved in these early hominins, implying some aspects of the life history strategy surprisingly similar to that of modern humans.

## Figures and Tables

**Table 1:** References and abbreviations of the feto-pelvic variables.

Variables	Abbreviation	References
maximal transverse diameter of the inlet	mati	[33]
inlet antero-posterior diameter	iap	[33]
inlet sacral breadth	isb	[34]
posterior inlet diameter	pi	[35]
pectineal angle	pa	[36]
midplane angle	ma	[36]
midplane antero-posterior diameter	map	[37]
interspinous diameter	isp	[33]
posterior midplane diameter	pm	[34]
midplane sacral breadth	msb	[36]
subpubic angle	spa	[38]
transverse outlet diameter	tout	[33]
posterior outlet diameter	po	[37]
outlet antero-posterior diameter	oap	[33]
sacral chord length	scl	[33]
sacral chord subtense	scs	[33]
inlet–midplane angle	ima	[33]
submentobregmatic diameter	smb	[33]
suboccipitobregmatic diameter	sob	[33]
suboccipitofrontal diameter	sof	[33]
mentovertical diameter	mv	[33]
mento-occipital diameter	moc	[33]
occipitofrontal diameter	of	[33]
biparietal diameter	bip	[33]
bitemporal diameter	bit	[33]
right tragion–bregma diameter	rtb	[39]
left tragion–bregma diameter	ltb	[39]
suboccipitobregmatic circumference	sobc	[39]
head circumference	hc	[39]
biacromial diameter	bia	[36]
sternum–thoracic vertebral diameter	stv	[33]
abdominal circumference	abdc	[36]
abdominal sagittal diameter	abds	[36]
transverse abdominal diameter	abdt	[36]
bitrochanterian diameter	bih	[36]
birthweight	wght	

**Table 2:** Capacity of the two canonical discriminant functions in predicting the three delivery outcomes.

Group	Predicted Group Membership			
	Caesarean	Spont. Vag. ^1^	Instrument	Total
Caesarean	19 (79%)	3 (13%)	2 (8%)	24
Spont. Vag.	4 (8%)	32 (63%)	15 (29%)	51
Instrument	5 (9%)	7 (12%)	44 (79%)	56

^1^ Spontaneous vaginal delivery.

**Table 3:** Capacity of the two canonical discriminant functions in predicting the three delivery outcomes and the predicted group membership of australopithecine “dyads”.

“Dyads”		Predicted Group Membership		
Pelvic Reconstructions	Head Size	Caesarean	Spont. Vag. ^1^	Instrument
A.L. 288-1 Lovejoy [10]	180 g	94%	5%	1%
A.L. 288-1 Lovejoy [10]	145 g	91%	8%	1%
A.L. 288-1 Lovejoy [10]	110 g	85%	15%	0%
A.L. 288-1 Haeusler [44]	180 g	77%	23%	0%
A.L. 288-1 Haeusler [44]	145 g	67%	33%	0%
A.L. 288-1 Haeusler [44]	110 g	44%	56%	0%
A.L. 288-1 Brassey [46]	180 g	96%	44%	0%
A.L. 288-1 Brassey [46]	145 g	93%	7%	0%
A.L. 288-1 Brassey [46]	110 g	84%	16%	0%
Sts 14 Haeusler [44]	180 g	71%	28%	1%
Sts 14 Haeusler [44]	145 g	61%	39%	0%
Sts 14 Haeusler [44]	110 g	37%	63%	0%
Sts 14 Berge [9] ^2^	180 g	92%	8%	0%
Sts 14 Berge [9] ^2^	145 g	88%	12%	0%
Sts 14 Berge [9] ^2^	110 g	83%	17%	0%
MH2 Schmid [50]	180 g	82%	18%	0%
MH2 Schmid [50]	145 g	75%	25%	0%
MH2 Schmid [50]	110 g	47%	53%	0%

^1^ Spontaneous vaginal delivery. ^2^ Supplementary canonical discriminant analysis without the following variables: midplane antero-posterior, midplane sacral breadth, posterior midplane, posterior outlet, outlet antero-posterior, sacral chord length, sacral chord subtense.

## Data Availability

All data are available in the main text. Further inquiries can be directed to the corresponding author.
